# Supplementary material for: The effect of heavy metals on thiocyanate biodegradation by an autotrophic microbial consortium enriched from mine tailings
Source: Appl Microbiol Biotechnol. 2020 Dec 2;105(1):417–27. doi: 10.1007/s00253-020-10983-4 (PMC7778618; doi:10.1007/s00253-020-10983-4)
Supplement: Supplementary file 1 — (PDF 285 kb). [file 253_2020_10983_MOESM1_ESM.pdf]

Applied Microbiology and Biotechnology

Supplementary Material

The effect of heavy metals on thiocyanate biodegradation by an autotrophic microbial consortium enriched from mine tailings

SHAFIEI[a], Farhad; WATTS[a], Mathew P.; PAJANK[a], Lukas; and MOREAU[a, b, *], John W.

[a] School of Earth Sciences, The University of Melbourne, Parkville, VIC 3010, Australia
[b] Currently at School of Geographical & Earth Sciences, University of Glasgow, Glasgow G12 8QQ, United Kingdom

*Corresponding author: john.moreau@gla.ac.uk
Telephone: +44 (0)141 330 5461
Fax: N/A

Supplementary Figures:

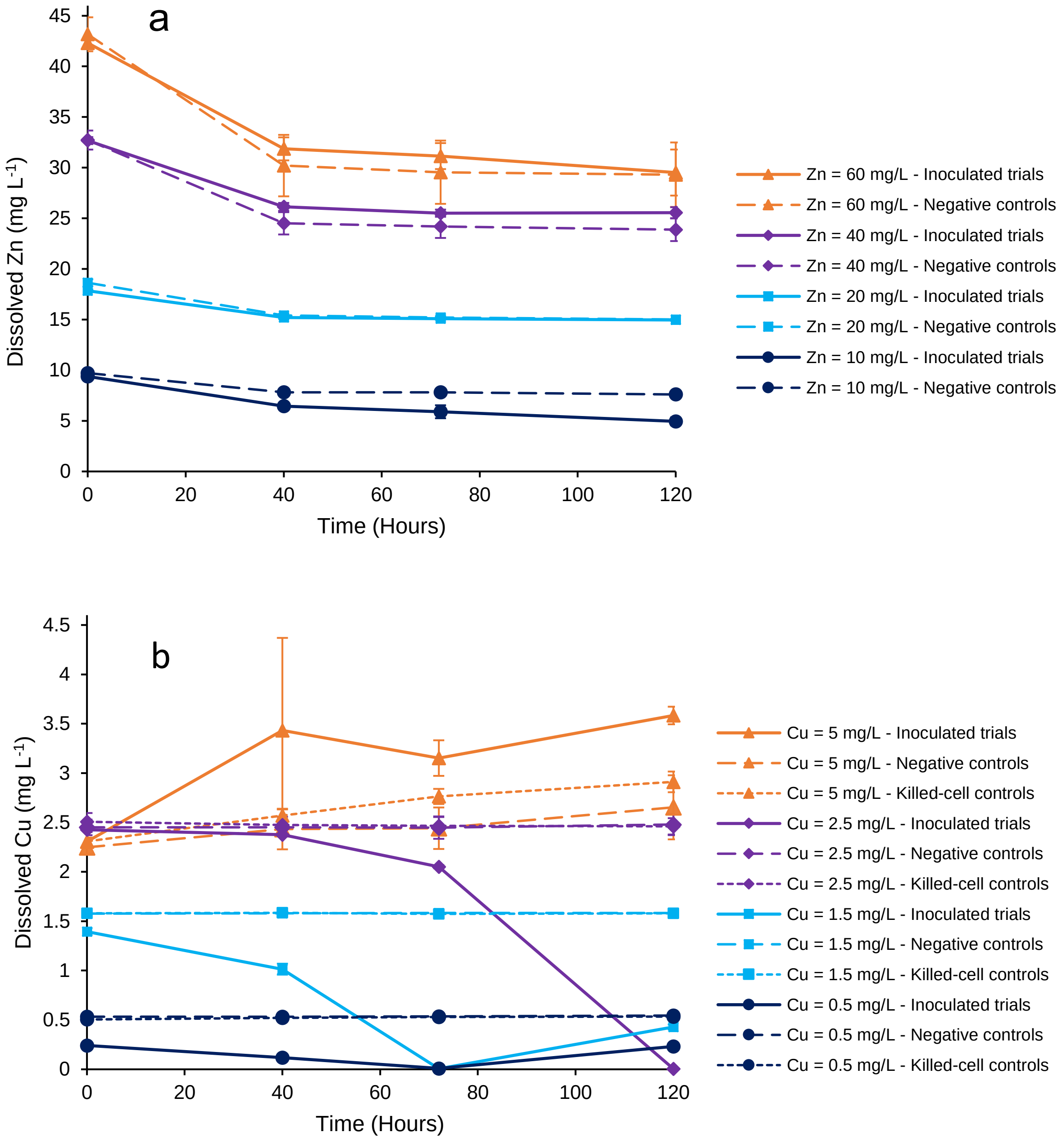

Figure S1 Change in dissolved metals concentrations in metal-amended trials; (**a**) Zn and (**b**) Cu; results represent mean ± standard deviation, n=3

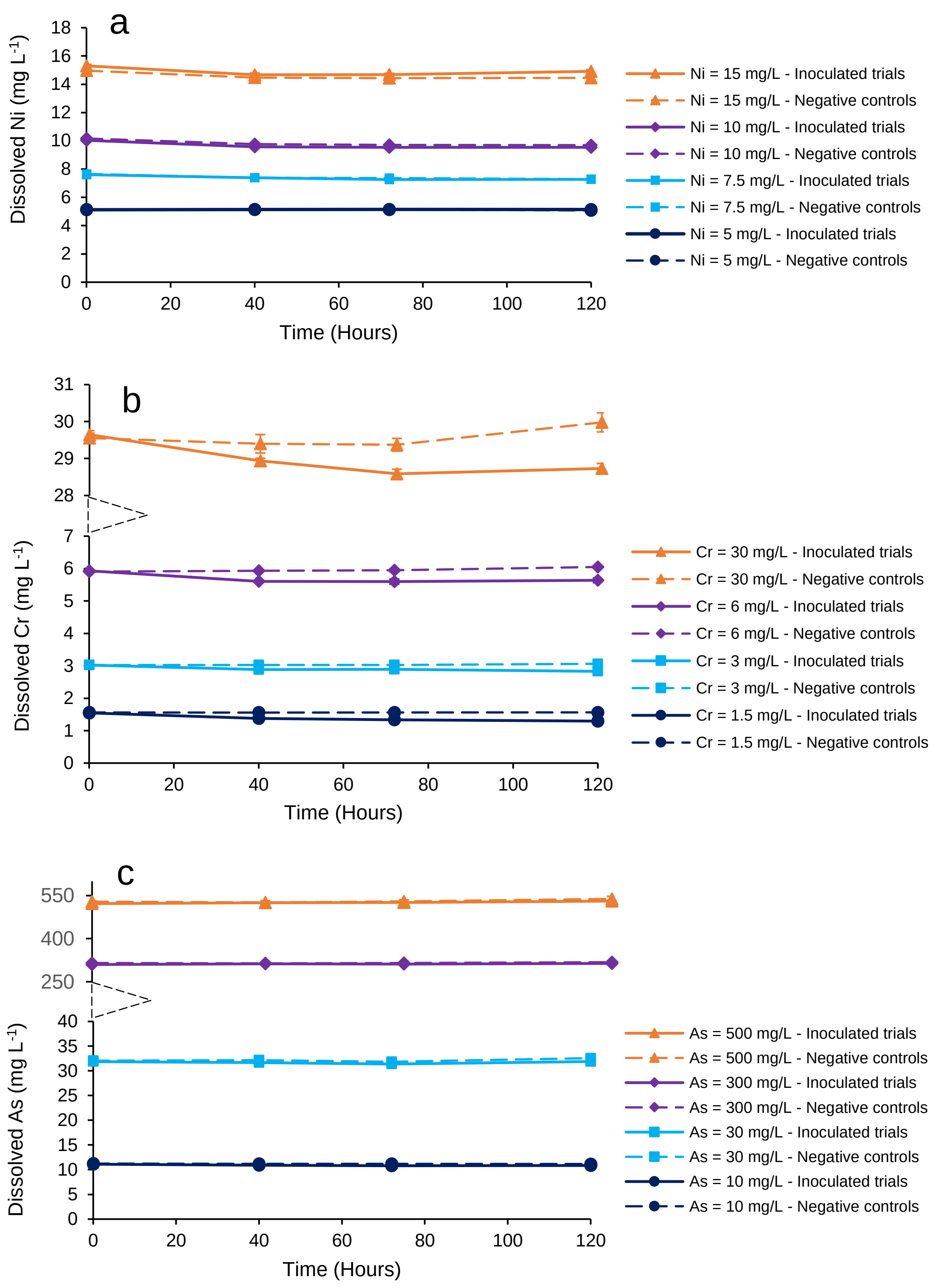

Figure S2 Change in dissolved metals concentrations in metal-amended trials; (**a**) Ni, (**b**) Cr, and (**c**) As; results represent mean ± standard deviation, n=3

Supplementary Tables:

Table S1 Distribution (molality) of predicted Zn species at different Zn amendment concentrations and non-amended controls

Zn species	Zn amendment concentration (mg L^{-1})				
	0	10	20	40	60
$ZnS(HS)^-$	2.83×10^{-6}	1.28×10^{-4}	2.54×10^{-4}	5.12×10^{-4}	7.76×10^{-4}
$Zn(HS)_3^-$	6.18×10^{-7}	2.71×10^{-5}	5.21×10^{-5}	9.76×10^{-5}	1.37×10^{-4}
$Zn(HS)_2$	3.95×10^{-8}	1.79×10^{-6}	3.56×10^{-6}	7.16×10^{-6}	1.08×10^{-5}
$ZnS(HS)_2^{2-}$	1.01×10^{-8}	4.43×10^{-7}	8.51×10^{-7}	1.60×10^{-6}	2.24×10^{-6}
$Zn(HS)_4^{2-}$	3.75×10^{-10}	1.59×10^{-8}	2.96×10^{-8}	5.17×10^{-8}	6.73×10^{-8}
$Zn(NTA)^-$	7.56×10^{-14}	3.54×10^{-12}	7.53×10^{-12}	1.75×10^{-11}	3.08×10^{-11}
$ZnOH(NTA)^{2-}$	5.32×10^{-16}	2.49×10^{-14}	5.30×10^{-14}	1.23×10^{-13}	2.17×10^{-13}
Zn^{2+}	5.15×10^{-16}	2.49×10^{-14}	5.31×10^{-14}	1.23×10^{-13}	2.17×10^{-13}
$ZnSO_4$	7.03×10^{-17}	3.40×10^{-15}	7.24×10^{-15}	1.68×10^{-14}	2.96×10^{-14}
$ZnCO_3$	5.03×10^{-17}	2.43×10^{-15}	5.17×10^{-15}	1.20×10^{-14}	2.11×10^{-14}
$ZnCl^+$	2.21×10^{-17}	1.08×10^{-15}	2.31×10^{-15}	5.43×10^{-15}	9.71×10^{-15}
$ZnOH^+$	2.02×10^{-17}	9.74×10^{-16}	2.07×10^{-15}	4.81×10^{-15}	8.48×10^{-15}
$ZnHCO_3^+$	1.72×10^{-17}	8.30×10^{-16}	1.77×10^{-15}	4.10×10^{-15}	7.22×10^{-15}
$ZnOHCl$	1.08×10^{-17}	5.25×10^{-16}	1.13×10^{-15}	2.65×10^{-15}	4.73×10^{-15}
$Zn(NTA)_2^{4-}$	5.51×10^{-18}	2.50×10^{-16}	5.32×10^{-16}	1.24×10^{-15}	2.18×10^{-15}
$Zn(SO_4)_2^{2-}$	4.36×10^{-18}	2.11×10^{-16}	4.49×10^{-16}	1.04×10^{-15}	1.84×10^{-15}
$ZnCl_2$	1.14×10^{-18}	5.60×10^{-17}	1.21×10^{-16}	2.88×10^{-16}	5.22×10^{-16}
$Zn(OH)_2$	7.59×10^{-19}	3.67×10^{-17}	7.82×10^{-17}	1.81×10^{-16}	3.20×10^{-16}
$ZnCl_3^-$	4.16×10^{-20}	2.06×10^{-18}	4.47×10^{-18}	1.08×10^{-17}	1.98×10^{-17}
$Zn(OH)_3^-$	2.80×10^{-21}	7.66×10^{-20}	2.88×10^{-19}	6.68×10^{-19}	1.18×10^{-18}
$ZnCl_4^{2-}$	1.54×10^{-21}	7.66×10^{-20}	1.68×10^{-19}	4.11×10^{-19}	7.64×10^{-19}
$Zn(OH)_4^{2-}$	1.75×10^{-25}	8.44×10^{-24}	1.80×10^{-23}	4.17×10^{-23}	7.35×10^{-23}
Total:	3.49×10^{-6}	1.57×10^{-4}	3.11×10^{-4}	6.18×10^{-4}	9.26×10^{-4}

Table S2 SI values of predicted Zn minerals at different Zn amendment concentrations and non-amended controls

Mineral phase	Chemical formula	Zn amendment concentration (mg L^{-1})				
		0	10	20	40	60
Bianchite	$ZnSO_4.6H_2O$	-16.75	-15.07	-14.74	-14.37	-14.13
Goslarite	$ZnSO_4.7H_2O$	-16.55	-14.86	-14.54	-14.17	-13.93
Smithsonite	$ZnCO_3$	-11.01	-9.33	-9.00	-8.64	-8.39
Sphalerite	ZnS	1.09	2.76	3.07	3.41	3.62
Wurtzite	ZnS	-1.38	0.29	0.60	0.93	1.15
Zincite	ZnO	-11.40	-9.72	-9.39	-9.02	-8.78
Zincosite	$ZnSO_4$	-22.20	-20.52	-20.19	-19.82	-19.58
$Zn(BO_2)_2$	$Zn(BO_2)_2$	-22.89	-21.21	-20.88	-20.51	-20.27
$Zn(OH)_2$	$Zn(OH)_2$	-12.53	-10.84	-10.51	-10.15	-9.90
$Zn(OH)_2$ (am)	$Zn(OH)_2$	-12.57	-10.88	-10.55	-10.19	-9.94
$Zn(OH)_2$ (beta)	$Zn(OH)_2$	-11.84	-10.16	-9.83	-9.46	-9.22
$Zn(OH)_2$ (epsilon)	$Zn(OH)_2$	-11.62	-9.94	-9.61	-9.25	-9.00
$Zn(OH)_2$ (gamma)	$Zn(OH)_2$	-12.06	-10.38	-10.05	-9.68	-9.44
$Zn_2(OH)_2SO_4$	$Zn_2(OH)_2SO_4$	-26.34	-22.97	-22.31	-21.58	-21.09
$Zn_2(OH)_3Cl$	$Zn_2(OH)_3Cl$	-25.00	-21.63	-20.97	-20.24	-19.74
$Zn_3(PO_4)_2.4H_2O$	$Zn_3(PO_4)_2.4H_2O$	-30.10	-25.05	-24.06	-22.97	-22.23
$Zn_3O(SO_4)_2$	$Zn_3O(SO_4)_2$	-55.51	-50.46	-49.48	-48.38	-47.64
$Zn_4(OH)_6SO_4$	$Zn_4(OH)_6SO_4$	-47.89	-41.15	-39.84	-38.38	-37.39
$Zn_5(OH)_8Cl_2$	$Zn_5(OH)_8Cl_2$	-58.45	-50.02	-48.38	-46.54	-45.30
$ZnCl_2$	$ZnCl_2$	-25.49	-23.80	-23.47	-23.09	-22.83
$ZnCO_3.H_2O$	$ZnCO_3.H_2O$	-10.80	-9.12	-8.79	-8.42	-8.18
Zn metal	Zn	-49.07	-47.38	-47.06	-46.69	-46.44
$ZnMoO_4$	$ZnMoO_4$	-11.95	-10.27	-9.94	-9.57	-9.33
ZnO (active)	ZnO	-11.26	-9.57	-9.24	-8.88	-8.63
ZnS (am)	ZnS	-1.27	0.40	0.72	1.05	1.27
$ZnSO_4.H_2O$	$ZnSO_4.H_2O$	-17.75	-16.06	-15.73	-15.37	-15.12

Table S3 Distribution (molality) of predicted Cu species at different Cu amendment concentrations and non-amended controls

Valence	Cu species	Cu amendment concentration (mg L^{-1})				
		0	0.5	1.5	2.5	5
Cu(I)	$Cu(S4)_2^{3-}$	1.16×10^{-12}	1.16×10^{-11}	3.26×10^{-11}	5.38×10^{-11}	1.08 × 10-10
	$CuS4S5^{3-}$	2.05×10^{-13}	2.04×10^{-12}	5.73×10^{-12}	9.46×10^{-12}	1.89×10^{-11}
	$CuCl_2^-$	8.87×10^{-25}	8.89×10^{-24}	2.53×10^{-23}	4.21×10^{-23}	8.66×10^{-23}
	CuCl	9.56×10^{-26}	9.57×10^{-25}	2.72×10^{-24}	4.53×10^{-24}	9.30×10^{-24}
	$CuCl_3^{2-}$	1.38×10^{-26}	1.38×10^{-25}	3.92×10^{-25}	6.55×10^{-25}	1.35×10^{-24}
	Cu^+	3.22×10^{-27}	3.22×10^{-26}	9.14×10^{-26}	1.52×10^{-25}	3.12×10^{-25}
Cu(II)	$Cu(HS)_3^-$	8.84×10^{-7}	8.79×10^{-6}	2.46×10^{-5}	4.04×10^{-5}	8.00×10^{-5}
	$Cu(NTA)^-$	4.71×10^{-21}	4.72×10^{-20}	1.34×10^{-19}	2.23×10^{-19}	4.57×10^{-19}
	$CuOH(NTA)^{2-}$	2.30×10^{-22}	2.30×10^{-21}	6.54×10^{-21}	1.09×10^{-20}	2.23×10^{-20}
	$Cu(NTA)_2^{4-}$	1.79×10^{-24}	1.79×10^{-23}	5.09×10^{-23}	8.48×10^{-23}	1.74×10^{-22}
	$CuCO_3$	1.17×10^{-24}	1.17×10^{-23}	3.32×10^{-23}	5.53×10^{-23}	1.13×10^{-22}
	$CuNH_3^{2+}$	2.59×10^{-25}	2.60×10^{-24}	7.36×10^{-24}	1.23×10^{-23}	2.51×10^{-23}
	Cu^{+2}	1.17×10^{-25}	1.17×10^{-24}	3.33×10^{-24}	5.54×10^{-24}	1.14×10^{-23}
	$CuOH^+$	1.13×10^{-25}	1.13×10^{-24}	3.22×10^{-24}	5.36×10^{-24}	1.10×10^{-23}
	$Cu(CO_3)_2^{2-}$	6.61×10^{-26}	6.61×10^{-25}	1.88×10^{-24}	3.13×10^{-24}	6.40×10^{-24}
	$CuSO_4$	1.70×10^{-26}	1.70×10^{-25}	4.83×10^{-25}	8.05×10^{-25}	1.65×10^{-24}
	$CuHCO_3^+$	7.78×10^{-27}	7.79×10^{-26}	2.21×10^{-25}	3.68×10^{-25}	7.55×10^{-25}
	$Cu(OH)_2$	6.87×10^{-27}	6.88×10^{-26}	1.95×10^{-25}	3.25×10^{-25}	6.66×10^{-25}
	CuH(NTA)	4.28×10^{-27}	4.29×10^{-26}	1.22×10^{-25}	2.03×10^{-25}	4.15×10^{-25}
	$CuCl^+$	3.24×10^{-27}	3.24×10^{-26}	9.21×10^{-26}	1.53×10^{-25}	3.15×10^{-25}
	$CuCl_2$	3.76×10^{-29}	3.77×10^{-28}	1.07×10^{-27}	1.79×10^{-27}	3.67×10^{-27}
	$Cu(OH)_3^-$	1.04×10^{-29}	1.04×10^{-28}	2.94×10^{-28}	4.90×10^{-28}	1.00×10^{-27}
	$CuCl_3^-$	1.72×10^{-32}	1.73×10^{-31}	4.91×10^{-31}	8.19×10^{-31}	1.69×10^{-30}
	$Cu(OH)_4^{2-}$	1.28×10^{-34}	1.28×10^{-33}	3.63×10^{-33}	6.05×10^{-33}	1.24×10^{-32}
	$CuCl_4^{2-}$	5.21×10^{-36}	5.22×10^{-35}	1.49×10^{-34}	2.48×10^{-34}	5.12×10^{-34}
Total:		*8.84×10^{-7}*	*8.79×10^{-6}*	*2.46×10^{-5}*	*4.04×10^{-5}*	*8.00×10^{-5}*

Table S4 SI values of predicted Cu minerals at different Cu amendment concentrations and non-amended controls

Mineral phase	Chemical formula	Cu amendment concentration (mg L^{-1})				
		0	0.5	1.5	2.5	5
Anilite	Cu0.25Cu1.5S	-9.53	-7.78	-6.98	-6.60	-6.06
Antlerite	$Cu_3(OH)_4SO_4$	-56.88	-53.88	-52.52	-51.86	-50.92
Atacamite	$Cu_2(OH)_3Cl$	-36.22	-34.22	-33.31	-32.87	-32.25
Azurite	$Cu_3(OH)_2(CO3)_2$	-54.19	-51.19	-49.83	-49.17	-48.23
BlaubleiI	Cu0.9Cu0.2S	1.45	2.55	3.05	3.29	3.63
BlaubleiII	Cu0.6Cu0.8S	-3.82	-2.42	-1.78	-1.48	-1.05
Brochantite	$Cu_4(OH)_6SO_4$	-72.70	-68.70	-66.88	-66.00	-64.75
Chalcanthite	$CuSO_4.5H_2O$	-25.54	-24.54	-24.08	-23.86	-23.55
Chalcocite	Cu_2S	-13.43	-11.43	-10.53	-10.08	-9.47
Chalcopyrite	$CuFeS_2$	10.38	11.38	11.84	12.06	12.37
Covellite	CuS	2.10	3.10	3.55	3.77	4.08
$Cu(OH)_2$	$Cu(OH)_2$	-18.48	-17.48	-17.03	-16.80	-16.49
Cu_2SO_4	Cu_2SO_4	-54.10	-52.10	-51.19	-50.75	-50.12
$Cu_3(PO_4)_2$	$Cu_3(PO_4)_2$	-57.60	-54.60	-53.24	-52.57	-51.64
$Cu_3(PO_4)_2.3H_2O$	$Cu_3(PO_4)_2.3H_2O$	-59.33	-56.33	-54.97	-54.31	-53.37
$CuCO_3$	$CuCO_3$	-19.20	-18.20	-17.75	-17.53	-17.22
Cu metal	Cu	-22.11	-21.11	-20.65	-20.43	-20.12
$CuMoO_4$	$CuMoO_4$	-18.71	-17.71	-17.26	-17.04	-16.72
$CuOCuSO_4$	$CuO.CuSO_4$	-48.03	-46.03	-45.12	-44.68	-44.05
Cuprite	Cu_2O	-36.15	-34.15	-33.24	-32.80	-32.18
$CuSO_4$	$CuSO_4$	-30.88	-29.88	-29.43	-29.21	-28.90
Djurleite	Cu0.066Cu1.868S	-12.68	-10.74	-9.87	-9.44	-8.85
Langite	$Cu_4(OH)_6SO_4.H_2O$	-75.07	-71.07	-69.26	-68.37	-67.13
Malachite	$Cu_2(OH)_2CO_3$	-35.59	-33.59	-32.68	-32.24	-31.61
Melanothallite	$CuCl_2$	-34.37	-33.37	-32.91	-32.69	-32.38
Nantokite	CuCl	-21.51	-20.51	-20.06	-19.84	-19.52
Tenorite	CuO	-17.42	-16.42	-15.97	-15.75	-15.44

Table S5 Distribution (molality) of predicted Ni species at different Ni amendment concentrations and non-amended controls

Ni species	Ni amendment concentration (mg L^{-1})				
	0	5	7.5	10	15
$Ni(NTA)^{-}$	2.10×10^{-6}	5.57×10^{-5}	5.60×10^{-5}	5.61×10^{-5}	5.61×10^{-5}
$Ni(NTA)_2^{4-}$	2.46×10^{-9}	1.76×10^{-10}	7.65×10^{-11}	4.89×10^{-11}	2.85×10^{-11}
Ni^{2+}	2.15×10^{-9}	2.14×10^{-5}	4.99×10^{-5}	7.86×10^{-5}	1.36×10^{-4}
$NiOH(NTA)^{2-}$	1.98×10^{-9}	5.27×10^{-8}	5.29×10^{-8}	5.30×10^{-8}	5.32×10^{-8}
$NiHCO_3^{+}$	2.80×10^{-10}	2.77×10^{-6}	6.46×10^{-6}	1.01×10^{-5}	1.75×10^{-5}
$NiSO_4$	2.67×10^{-10}	2.65×10^{-6}	6.18×10^{-6}	9.71×10^{-6}	1.68×10^{-5}
$NiNH_3^{2+}$	2.59×10^{-10}	2.57×10^{-6}	6.00×10^{-6}	9.45×10^{-6}	1.64×10^{-5}
$NiCO_3$	1.36×10^{-10}	1.35×10^{-6}	3.14×10^{-6}	4.93×10^{-6}	8.50×10^{-6}
$NiCl^{+}$	1.03×10^{-10}	1.03×10^{-6}	2.40×10^{-6}	3.79×10^{-6}	6.58×10^{-6}
$NiOH^{+}$	1.03×10^{-11}	1.03×10^{-7}	2.39×10^{-7}	3.77×10^{-7}	6.52×10^{-7}
$Ni(NH_3)_2^{2+}$	5.81×10^{-12}	5.78×10^{-8}	1.35×10^{-7}	2.12×10^{-7}	3.67×10^{-7}
$Ni(OH)_2$	2.00×10^{-13}	1.99×10^{-9}	4.64×10^{-9}	7.30×10^{-9}	1.26×10^{-8}
$Ni(SO_4)_2^{2-}$	6.31×10^{-14}	6.26×10^{-10}	1.46×10^{-9}	2.30×10^{-9}	3.97×10^{-9}
$NiCl_2$	1.21×10^{-14}	1.21×10^{-10}	2.83×10^{-10}	4.47×10^{-10}	7.79×10^{-10}
$Ni(OH)_3^{-}$	1.47×10^{-16}	1.46×10^{-12}	3.41×10^{-12}	5.37×10^{-12}	9.29×10^{-12}
Total:	*2.10×10^{-6}*	*8.77×10^{-5}*	*1.31×10^{-4}*	*1.73 × 10-4*	*2.59×10^{-4}*

Table S6 SI values of predicted Ni minerals at different Ni amendment concentrations and non-amended controls

Mineral phase	Chemical formula	Ni amendment concentration (mg L^{-1})				
		0	5	7.5	10	15
Bunsenite	NiO	-5.86	-1.86	-1.49	-1.30	-1.06
Morenosite	$NiSO_4.7H_2O$	-9.79	-5.79	-5.42	-5.23	-4.99
$Ni(OH)_2$	$Ni(OH)_2$	-6.22	-2.22	-1.86	-1.66	-1.42
$Ni3(PO_4)_2$	$Ni_3(PO_4)_2$	-14.35	-2.36	-1.26	-0.67	0.04
$Ni_4(OH)_6SO_4$	$Ni_4(OH)_6SO_4$	-25.00	-9.02	-7.54	-6.76	-5.81
$NiCO_3$	$NiCO_3$	-7.45	-3.45	-3.08	-2.89	-2.65
$NiMoO_4$	$NiMoO_4$	-4.35	-0.35	0.01	0.21	0.45
NiS (alpha)	NiS	1.95	5.94	6.31	6.51	6.75
NiS (beta)	NiS	7.45	11.44	11.81	12.01	12.25
NiS (gamma)	NiS	9.15	13.14	13.51	13.71	13.95
Retgersite	$NiSO_4.6H_2O$	-9.87	-5.87	-5.51	-5.31	-5.07

Table S7 Distribution (molality) of predicted Cr species at different Cr amendment concentrations and non-amended controls

Species	Cr amendment concentration (mg L^{-1})				
	0	1.5	3	6	30
CrO_4^{2-}	0	1.10×10^{-5}	2.21×10^{-5}	4.41×10^{-5}	2.20×10^{-4}
$NaCrO_4^-$	0	1.38×10^{-6}	2.76×10^{-6}	5.52×10^{-6}	2.75×10^{-5}
$HCrO_4^-$	0	3.87×10^{-7}	7.74×10^{-7}	1.55×10^{-6}	7.72×10^{-6}
$KCrO_4^-$	0	1.99×10^{-7}	3.99×10^{-7}	8.05×10^{-7}	4.33×10^{-6}
$CrO_3HPO_4^{2-}$	0	4.71×10^{-11}	9.43×10^{-11}	1.89×10^{-10}	9.44×10^{-10}
$Cr_2O_7^{2-}$	0	9.78×10^{-12}	3.91×10^{-11}	1.56×10^{-10}	3.91×10^{-9}
$CrO_3SO_4^{2-}$	0	1.18×10^{-14}	2.36×10^{-14}	4.72×10^{-14}	2.36×10^{-13}
H_2CrO_4	0	5.48×10^{-15}	1.10×10^{-14}	2.19×10^{-14}	1.09×10^{-13}
CrO_3Cl^-	0	1.66×10^{-15}	3.31×10^{-15}	6.61×10^{-15}	3.30×10^{-14}
$CrO_3H_2PO_4^-$	0	1.45×10^{-16}	2.91×10^{-16}	5.81×10^{-16}	2.90×10^{-15}
Total:	*0*	1.30×10^{-5}	2.60×10^{-5}	5.20×10^{-5}	2.60×10^{-4}

Table S8 SI values of predicted Cr minerals at different Cr amendment concentrations and non-amended controls

Mineral phase	Chemical formula	Cr amendment concentration (mg L^{-1})				
		0*	1.5	3	6	30
$(NH_4)_2CrO_4$	$(NH_4)_2CrO_4$	-	-10.09	-9.79	-9.49	-8.80
$CaCrO_4$	$CaCrO_4$	-	-5.62	-5.32	-5.02	-4.33
CrO_3	CrO_3	-	-17.57	-17.27	-16.96	-16.27
$CuCrO_4$	$CuCrO_4$	-	-25.32	-25.02	-24.72	-24.02
$K_2Cr_2O_7$	$K_2Cr_2O_7$	-	-13.26	-12.66	-12.05	-10.59
$K2CrO_4$	K_2CrO_4	-	-9.02	-8.72	-8.41	-7.64
$MgCrO_4$	$MgCrO_4$	-	-13.38	-13.07	-12.77	-12.08
$Na_2Cr_2O_7$	$Na_2Cr_2O_7$	-	-19.01	-18.41	-17.81	-16.41
Na_2CrO_4	Na_2CrO_4	-	-10.92	-10.62	-10.32	-9.62

* None of Cr minerals were predicted in non-amended trials of Cr tolerance experiment

Table S9 SI values for predicted As minerals at different As amendment concentrations and non-amended controls

Species	As amendment concentration (mg L^{-1})				
	0	10	30	300	500
$HAsO_4^{2-}$	0	6.84×10^{-5}	2.05×10^{-4}	2.06×10^{-3}	3.43×10^{-3}
$H_2AsO_4^{-}$	0	3.91×10^{-6}	1.17×10^{-5}	1.13×10^{-4}	1.82×10^{-4}
AsO_4^{3-}	0	8.08×10^{-8}	2.44×10^{-7}	2.62×10^{-6}	4.59×10^{-6}
H_3AsO_4	0	9.50×10^{-12}	2.84×10^{-11}	2.69×10^{-10}	4.31×10^{-10}
Total:	*0*	*7.24×10^{-5}*	*2.17×10^{-4}*	*2.17×10^{-3}*	*3.62×10^{-3}*

Table S10 SI values of predicted As minerals at different As amendment concentrations and non-amended controls

Phase	Chemical formula	As amendment concentration (mg L^{-1})				
		0	10	30	300	500
$AlAsO_4.2H_2O$	$AlAsO_4.2H_2O$	-	-8.71	-8.24	-7.26	-7.06
As_2O_5	As_2O_5	-	-28.66	-27.71	-25.76	-25.35
$Ca_3(AsO_4)_2.4H_2O$	$Ca_3(AsO_4)_2.4H_2O$	-	-5.85	-4.90	-2.97	-2.57
$Co_3(AsO_4)_2$	$Co_3(AsO_4)_2$	-	-12.21	-11.26	-9.33	-8.93
$Cu_3(AsO_4)_2.2H_2O$	$Cu_3(AsO_4)_2.2H_2O$	-	-58.02	-57.07	-55.07	-54.62
$Mn_3(AsO_4)_2.8H_2O$	$Mn_3(AsO_4)_2.8H_2O$	-	-7.25	-6.31	-4.42	-4.05
$Ni_3(AsO_4)_2.8H_2O$	$Ni_3(AsO_4)_2.8H_2O$	-	-18.84	-17.89	-15.96	-15.56
$Zn_3(AsO_4)_2.2.5H_2O$	$Zn_3(AsO_4)_2.2.5H_2O$	-	-36.65	-35.69	-33.71	-33.28

* None of As minerals were predicted in non-amended trials of As tolerance experiment
